# Microbiological evaluation of the efficacy of two new biodetergents on multidrug-resistant nosocomial pathogens

**DOI:** 10.1186/1476-0711-8-35

**Published:** 2009-12-16

**Authors:** Giorgio Liguori, Maria Bagattini, Francesca Gallè, Valeria Quartucci, Valeria Di Onofrio, Mario Negrone, Maria Triassi

**Affiliations:** 1 Cattedra di Igiene ed Epidemiologia, Università degli studi di Napoli "Parthenope", Napoli, Italy; 2 Dipartimento di Scienze Mediche Preventive, Sezione di Igiene, Università degli studi di Napoli "FedericoII", Napoli, Italy; 3 Servizio di Igiene degli alimenti e della nutrizione, Dipartimento di Medicina Preventiva, Agenzia di Sanità Pubblica, Potenza, Italy

## Abstract

**Background:**

In the last few years, several outbreaks of nosocomial infections caused by multidrug-resistant pathogenic agents have been observed, and various biocides products were developed in order to control this phenomenon. We investigated the efficacy of two natural biodetergents composed of plants and kelps extracts, BATT1 and BATT2, against multidrug-resistant strains.

**Methods:**

*In-vitro *antibacterial efficacy of BATT1 and BATT2 against nosocomial multidrug-resistant isolates was assessed using a suspension-inhibition test, with and without bovine serum albumin (BSA). The test was also carried out on glass surfaces with and without BSA.

**Results:**

*In vitro *tests with both biocidal disinfectants at 25% concentration demonstrated an overall drop in bacterial, mould and yeast counts after 10 min of contact with or without organic substances. For *Pseudomonas aeruginosa*, it was necessary to use undiluted disinfectants with and without an organic substance. The same results were obtained in tests carried out on glass surfaces for all strains.

**Conclusions:**

The natural products BATT1 and BATT2 behave like good biocides even in presence of organic substances. The use of both disinfectants may be beneficial for reducing hospital-acquired pathogens that are not susceptible to disinfectants.

However, it has to be stressed that all these experiments were carried out *in vitro *and they still require validation from use in clinical practice.

## Introduction

At present, biocides are an integral component of clinical medicine, and serve to prevent the dissemination of nosocomial pathogens in the hospital environment [1]. In the last few years, despite remarkable progress in our knowledge of risk factors, prevention and control measures, the incidence of nosocomial infections has not decreased, and many outbreaks have been caused by new multidrug-resistant pathogens that have been selected by excessive and often irrational use of antibiotics [2,3]. These microorganisms are resistant to the majority of antibiotics and to many disinfectants, which has resulted in an increase in environmental contamination [4,5]. In many cases, it has been demonstrated that the molecular mechanisms responsible for antibiotic resistance are the same as those implicated in lack of susceptibility to biocides; anyway, some biocides have the ability to select for antibiotic resistant strains and vice versa [6-10]. Also, organic contamination reduces the effectiveness of disinfectants and antiseptics that are used extensively in medical and healthcare facilities for the disinfection of mucous membranes and wounds, and for the sterilization of medical instruments and equipment surfaces that are often contaminated with organic materials. The influence of such materials on the practical use of disinfectants should not be ignored [11].

In the last few years, following the increased circulation of pathogens that are not susceptible to several disinfectants, many biocides and numerous *in vitro *tests have been developed to assess the effectiveness of these products in specific clinical applications [12]. NTI 30 C4281 BATT1 and NTI 30 C4282 BATT2 (Natural Technologies Italia srl), referred to here as BATT1 and BATT2, are two new natural biocides/detergents formulated with seaweed and plant extracts and synergistic blends of surfactants derived from kelp and other plants of low biological toxicity. Alkyl-amino carboxylate is the active principle in both products.

The aim of the present study was to assess the efficacy of these biocides/detergents against multidrug-resistant nosocomial bacteria.

## Materials and methods

### Disinfectants

Solutions of BATT1 and BATT2 disinfectants were prepared by dilution in sterile water at 10, 15, 20 and 25%. The bactericidal effects of BATT1 and BATT2 on multidrug-resistant nosocomial pathogens were assessed using a suspension-inhibition test as recommended by the European Committee for Standardization (CEN) with or without bovine serum albumin (BSA) 0.3 g/100 ml [13]. The bactericidal effects of disinfectants were assessed using a slightly modified suspension-inhibition test as recommended by the CEN. The bactericidal effects of BATT1 and BATT2 on reference strains were tested from manufacturer (data not showed).

### Culture methods

Environmental strains (*Acinetobacter baumannii, Pseudomonas aeruginosa*, methicillin-resistant *Staphylococcus epidermidis*, methicillin-resistant *Staphylococcus aureus*, high-level aminoglycoside-resistant *Enterococcus faecalis*, extended-spectrum beta-lactamase (ESβL)-producing *Klebsiella pneumoniae, Candida albicans, Aspergillus fumigatus *and *Legionella pneumophila ser.1*) were isolated from 2002 to 2005 in the intensive care units (ICUs) of the University Hospital "Federico II" in Naples, Italy, during sanitation checks following nosocomial outbreaks. These microorganisms are recognized as the major hospital-acquired pathogens that cause severe infections such as pneumonia, septicaemia, urinary tract infections and surgical site infections. Throughout this study, the isolates per species were of the same clonal type, showing identical macrorestriction PFGE pattern (data not showed).

Swabs moistened with Brain-Heart Infusion broth were used to sample horizontal surfaces and points of frequent hand contact, as well as monitoring equipment, drug trolleys, respirators and sinks. Contact plates (Rodac; International PBI) with selective agar were used to sample other surfaces (floor, walls and beds), and air was sampled through the Surface Air System (International PBI). Culture specimens were enriched overnight at 37°C in Brain-Heart Infusion broth and then subcultivated on agar plates.

Isolates were identified by a commercial microidentification system (API 20E; bioMèrieux, Marcy-L'Etoile, France).

Environmental *Legionella *isolates were obtained from multiple sites in patients' rooms. For each outlet, 2 l of hot water was collected in a sterile bottle that contained 1 ml of a 10 mg/ml solution of sodium thiosulphate. The water temperature and residual free chlorine were determined immediately after collection. Samples were concentrated by filtration through cellulose acetate membrane filters (0.22 μm pore size) and resuspended into 10 ml of the filtrate. Aliquots of the suspension were plated on to Buffered Charcoal Yeast Extract (BCYE) agar with Legionella Glycine, Vancomycin, Polymyxin B, Cycloheximide, and Wadowsky and Yee selective supplements (Oxoid, Basingstoke, UK). Plates were incubated in 2.5% CO_2 _for 5 days at 37°C and examined daily for evidence of growth. Gram-negative typical colonies that required L-cysteine for growth were harvested, centrifuged at 3000 rpm for 20 min and resuspended in sterile distilled water prior to serological identification.

Resistance patterns of these microorganisms have been determined as following.

### Susceptibility testing and screening

Susceptibility tests of *K. pneumoniae, A. baumannii *and *P. aeruginosa *were performed using the BD Phoenix (Phoenix Technologies Ltd, San Jose, CA, USA) system (MIC/panel susceptibility card) according to the manufacturer's instructions (*E. coli *25922 ATCC QC, *P. aeruginosa *ATCC QC 27853, *E. coli *ATCC QC 35218)

ESβL activity in *K. pneumoniae *was evaluated using the double-disc synergy test between cephalosporins or monobactam and clavulanate disks on Muller Hinton agar plates. ESβL activity was confirmed by Etest cefotaxime/cefotaxime+clavulanic and ceftazidime/ceftazidime + clavulanic acid strip as recommended by the manufacturer (*K. pneumoniae *ATCC QC 700603).

High-level aminoglycoside resistance in *E. faecalis *was determined in 96-well microtitre plates using Gentamicin and streptomycin. For QC of HLAR screen tests was used *E. faecalis *ATCC 29212.

Methicillin and vancomycin were used to test for resistance in *Staphylococcus *spp. by antibiotic disc diffusion method (*S. aureus *MRSA ATCC QC 43300).

Susceptibility testing of yeast and mould was detected by E-test using *C. albicans *ATCC QC 90028 and *A. fumigatus ATCC *204305.

Finally, MICs for *L. pneumophila *were determined using broth microdilution in liquid BCYE medium with selective supplement (*L. pneumophila ser.1 *ATCC QC 33152).

Throughout this study, results were interpreted according to the Clinical and Laboratory Standards Institute (CLSI) criteria for broth microdilution and disk diffusion methods [14].

Antimicrobial susceptibility testing showed common multidrug-resistant antibiotypes for all the isolated microorganisms (Table 1). In particular, the antimicrobial susceptibility patterns of the ESβL-producing *K. pneumoniae *showed resistance to penicillins, monobactams and third-generation cephalosporins. Also the *K. pneumoniae *strain was identified as ESβL-producer by Etest analysis (Table 2). The antimicrobial susceptibility analysis showed high-level aminoglycoside-resistance in *E. faecalis *and methicillin-resistance in *Staphylococcus *spp. that were either coagulase positive or negative (data not showed).

**Table 1:** Antimicrobial susceptibility patterns of strains tested (*Etest analysis).

ANTIBIOTIC	*K. pneumoniae*	*A.baumannii*	*P. aeruginosa*	*E. faecalis*	*C. albicans*	*A. fumigatus*	*L. pneumophila*
Amikacin	<4	>32	>32				

Amoxicillin-clavulanate	>16/8	>16	>32				

Ampicillin	>16		>32				

Ampicillin-sulbactam	>16/8						

Aztreonam	>16						

Cefazolin	>16		>64				

Cefepime			2				

Cefotaxime	>16	>32	>64				

Ceftazidime	>32	>16	>16				

Ceftriaxone	>32						

Chloramphenicol	16	>16					

Ciprofloxacin	>2	>2					>2

Doxycycline							>8

Erythromycin							1

Gentamicin	≤ 2	>8	>8				

Gentamicin high-level				>2000			

Imipenem	≤ 1	>8	>8				

Levofloxacin	≤ 1	>2	>2				>2

Meropenem	≤ 1	>8	>8				

Netilmicyn high-level				>2000			

Nitrofurantoine	≤ 16		>512				

Norfloxacine	≤ 2						

Piperacillin	>64	>64	16				

Piperacillin-tazobactam	≥ 64/4	≥ 64/4	16				

Streptomycin high-level				>2000			

Tetracycline	>8	>8					

Trimethoprim-sulfamethoxazole	≤ 0.5/9.5	>2/38					

5-Fluorocytosine					>32*		

Fluconazole					>64*		

Itraconazole					>1*	>1*	

Amphotericin B					>1*	>2*	

**Table 2:** MIC values of *K. pneumoniae *strain identified as ESβL-producer (Etest analysis).

	MIC of drug (mg/L)			
*K. pneumoniae*	CTX	CTX+CLA	CAZ	CAZ+CLA

MIC	>16	0.023	>32	0.125

CAZ: ceftazidime; CLA: clavulanic acid; CTX: cefotaxime

### In vitro quantitative suspension tests

Microbial suspensions in Luria-Bertani broth were prepared from fresh cultures of the above microorganisms, and subcultivated in non-selective media (Brain-Heart Infusion agar for Gram-negative bacteria, *Enterococcus *and *Candida *spp., Tryptone Soy Agar for *Staphylococcus *spp., Columbia Blood Agar for *Aspergillus fumigatus*). *L. pneumophila *was cultivated on a selective medium, BCYE Agar with selective supplements.

Final concentrations of inocula of 10^8 ^CFU/ml bacteria and 10^6^CFU/mL yeasts and moulds were measured using a spectrophotometer (UV/VIS Lambda 2, PerkinElmer, Monza, Italy) (OD_660 _0.08). A 100-μl suspension for each test was added to tubes that contained 900 μl of sterile physiological solution (control) or 900 μl of each disinfectant solution at different concentrations (10, 15, 20 and 25%), and left for 5, 10 and 15 min at 20 ± 2°C in a thermostatic bath. Each inoculum was prepared twice, with and without BSA 0.3 g/100 ml.

After incubation, 100 μl were removed from each inoculum and smeared on Bacto D/E Neutralizing agar (Becton Dickinson) after serial dilution (1:10,1:100,1:1000,1:10000) in phosphate buffered saline (PBS ph 7.4), in order to count the number of colonies surviving on each plate at different dilution. According to the standards, an efficacious biocide must reduce the initial count by 4 or 5 log units, and the efficacy is estimated by the ratio between the number of microorganisms in the starting solution (inocula of 10^8 ^CFU/ml bacteria and 10^6 ^CFU/mL yeasts and moulds) and number of colonies surviving on neutralization plates. Plates were incubated at 37°C for 24 h for bacterial strains and at 32°C for 72 h for *C. albicans *and *A. fumigatus *strains. Only the aliquots of the reaction mixture (100 μl) that contained *L. pneumophila *cells and disinfectants after incubation were added to 900 μl of neutraliser solution and left at 20 ± 2°C for 3 min. After serial dilution as above, the aliquots were smeared on BCYE agar with supplements. These plates were incubated at 37°C in 2.5% CO_2 _for 5 days. The test was also carried out on glass surfaces initially contaminated with the microbial inocula, prepared as above, for which the same concentrations were used as for the suspension test, with and without BSA. After 5, 10 and 15 min of sanitation with both disinfectant solutions at different concentration (10, 15, 20 and 25%), the surfaces were rinsed with a sterile physiological solution, and 100 μl was removed from the rinsing solution and smeared on D/E Neutralizing agar after serial dilution. Plates were incubated under the same conditions as above. For *L. pneumophila*, it was only necessary to use the neutraliser solution and selective medium. The experiments were repeated three times on different days.

## Results

### In vitro quantitative suspension tests

Bacterial, mould and yeast concentration was evaluated by colony counts on agar plates (Bacto D/E Neutralizing agar, BCYE agar with supplements) after incubation under the described conditions.

The results of the *in vitro *tests were interpreted in accordance with the CEN Standards [13]. According to the standards, an efficacious biocide must reduce the initial count by 4 or 5 log units, and the efficacy is estimated by the ratio between the number of microorganisms in the starting solution (inocula of 10^8 ^CFU/ml bacteria and 10^6^CFU/mL yeasts and moulds) and number of colonies surviving on neutralization plates.

Bactericidal activity of BATT1 and BATT2 was observed at a concentration of 25% after 10 min contact. *In vitro *tests with BATT1 and BATT2 demonstrated an overall drop in microbial counts, without BSA (Table 3). The same results were obtained in presence of BSA. To reduce *P. aeruginosa *count, it was necessary to use 50%, 75% concentrations and finally both undiluted disinfectants, with and without BSA (data not shown). The same results were obtained in the tests carried out on glass surfaces for all nosocomial strains. Also in this test the biocide effect on *P. aeruginosa *was obtained by using undiluted disinfectants with and without an organic substance.

**Table 3:** Experimental conditions and microbicidal effect of BATT1 e BATT2 on examined microorganisms

Microorganism	BATT1-BATT2 concentration	Contact time (min)	Temp (C°)	Organic load (BSA g/100 ml)	Test methodology	Inoculum (log_10_)	Biocidal activity (log_10 reduction_) without BSA	Biocidal activity (log_10 reduction_) with BSA
*Acinetobacter baumannii*	25%	10	20 ± 2	0.3	Suspension Carrier	10^8 ^CFU/ml	8	8

*Pseudomonas aeruginosa*	undiluted disinfectants	10	20 ± 2	0.3	Suspension Carrier	10^8 ^CFU/ml	8	8

MR *Staphylococcus epidermidis*	25%	10	20 ± 2	0.3	Suspension Carrier	10^8 ^CFU/ml	8	8

MR *Staphylococcus aureus*	25%	10	20 ± 2	0.3	Suspension Carrier	10^8 ^CFU/ml	8	8

ESβL-producing *Klebsiella pneumoniae*	25%	10	20 ± 2	0.3	Suspension Carrier	10^8 ^CFU/ml	8	8

*Candida albicans*	25%	10	20 ± 2	0.3	Suspension Carrier	10^6 ^CFU/mL	6	6

*Aspergillus fumigatus*	25%	10	20 ± 2	0.3	Suspension Carrier	10^6 ^CFU/mL	6	6

HLAR *Enterococcus faecalis*	25%	10	20 ± 2	0.3	Suspension Carrier	10^8 ^CFU/ml	8	8

*Legionella pneumophila ser.1*	25%	10	20 ± 2	0.3	Suspension Carrier	10^8 ^CFU/ml	8	8

## Discussion

Biodetergents BATT1 and BATT2 are natural products that act as disinfectants and cleaning agents. In our experience, this formulation from seaweed and plant extracts had beneficial and effective results. *In vitro *tests showed an overall drop in bacterial, mould and yeast counts after 10 min contact, with or without BSA, when the disinfectants were used at a 25% concentration. These products are not consumed by organic residues, which normally reduce the activity of oxidising disinfectants such as chlorine and ozone. The disinfectant effect of chlorine derivatives and ozone occurs when the oxidation of organic substances has been achieved, which implies the need for higher-than-standard concentrations. However, increasing the oxidising disinfectant concentration is not entirely without risk. Evidence for the development of reduced susceptibility caused by excessive exposure to disinfectants, including quaternary ammonium compounds and bis-biguanides, has been reported [15,16]. The use of BATT1 and BATT2 disinfectants, especially if alternate, may be beneficial for reducing disinfectant-non-susceptible microbes.

In the last few years, we have observed outbreaks of nosocomial infections caused by multidrug-resistant pathogens that have been selected by excessive and often irrational use of antibiotics and large use of disinfectants [2,5]. Following increased circulation of pathogens that are non-susceptible to several disinfectants, numerous *in vitro *tests have been developed to assess the effectiveness of various biocides in specific clinical situations [12]. In the present study, the efficacy of the two natural biocides/detergents BATT1 and BATT2 was assessed againstmultidrug-resistant strains isolated in the ICUs of University Hospital "Federico II" in Naples during nosocomial outbreaks. BATT1 and BATT2 disinfectants seem to be efficacious and cost-effective and may be useful in the control of microbial contamination in hospital settings.

It has to be stressed, however, that the reported experiments were carried out *in vitro *and have only a predictive significance. The efficacy demonstrated by these surrogate testing methods still requires validation from clinical practice.

## Competing interests

The authors declare that they have no competing interests.

## Authors' contributions

GL conceived of the study and carried out its design. MB performed the assays and drafted the manuscript. FG and VDO drafted and edited the manuscript. VQ contributed to the assays performance. MN analyzed the results of tests. MT participated in the design and supervision of the study.

All authors have read and approved the final manuscript.
